# Case Report: Visual Deprivation in Pusher Syndrome Complicated by Hemispatial Neglect After Basal Ganglia Stroke

**DOI:** 10.3389/fneur.2021.706611

**Published:** 2021-09-22

**Authors:** Qian Zhang, Lixia Zhang, Wei He, Xuemei Zheng, Zhengrui Zhao, Yuanli Li, Shutian Xu, Juan Zheng, Xin Zhuang, Wenting Jia, Chengyuan Zhu, Hua Xu, Chunlei Shan, Wenhua Chen, Jingpu Zhao, Sijing Chen

**Affiliations:** ^1^Rehabilitation Department, The Geriatric Hospital Affiliated to Nanjing Medical University, Nanjing, China; ^2^Rehabilitation Department, Rehabilitation Hospital Affiliated to Nanjing Institute of Physical Education, Wuxi, China; ^3^Rehabilitation Department, Xinjiang Korla Bazhou People's Hospital, Korla, China; ^4^School of Rehabilitation Science, Shanghai University of Traditional Chinese Medicine, Shanghai, China; ^5^Engineering Research Center of Traditional Chinese Medicine Intelligent Rehabilitation, Ministry of Education, Shanghai, China; ^6^Center of Rehabilitation Medicine, Yueyang Hospital of Integrated Traditional Chinese and Western Medicine, Shanghai, China; ^7^Rehabilitation Department, The First People's Hospital Affiliated With Shanghai Jiao Tong University, Shanghai, China; ^8^Rehabilitation Department, The First Affiliated Hospital of Shenzhen University, Shenzhen, China; ^9^Rehabilitation Department, The First Affiliated Hospital of Nanjing Medical University, Nanjing, China

**Keywords:** case report, hemispatial neglect, pusher syndrome, visual deprivation, basal ganglia and temporal lobe, stroke

## Abstract

We aimed to explore whether motor function and activities of daily life (ADL) could be improved with the application of visual deprivation in two patients with Pusher syndrome complicated by hemispatial neglect after right basal ganglia stroke. We assessed two stroke patients suffering from severe motor disturbances, both tilting heavily to the left, with diagnoses of Pusher syndrome and left hemispatial neglect. Vision in the left eye was deprived using patches during clinical rehabilitation. Motor function promotion was confirmed using the Burke Lateropulsion Scale (BLS), Fugl–Meyer Balance Scale (FMBS), and Holden grade (HG), while the Barthel index (BI) assessed ADL immediately and 1 week after intervention. Both patients regained standing balance immediately using visual deprivation, as well as walking ability, although both scored 0 on the FMBS and HG. After 1 week of treatment, one patient increased to 11 and 3 on the FMBS and HG, respectively, while the BLS score decreased from 12 to 2, and the ADL increased from 23 to 70. The other patient demonstrated increases to 10 and 3 on the FMBS and HG, respectively, with the BLS decreasing from 13 to 3, and the ADL increasing from 25 to 60. Therefore, in the rehabilitation treatment of Pusher syndrome complicated by hemispatial neglect due to basal ganglia stroke, visual deprivation can significantly improve motor function and shorten the treatment course.

## Introduction

Pusher syndrome (PS), also known as “tilt syndrome,” is a severe postural control disorder that occurs after stroke and is characterized by uncorrectable balance dysfunction. The patient tilts strongly to the hemiplegic side in both sitting and standing positions and resists external forces that move the body to the healthy side. The typical clinical manifestations are (a) left-sided paralysis with left-sided hemiplegic visual–spatial neglect; (b) weight-bearing on the left hip and shortening of the right trunk in the sitting position; (c) difficulty in bed and chair transfer, especially to the chair on the healthy side; (d) the center of gravity is skewed to the left when standing, making it difficult to maintain a balanced stance; (e) the left lower limb is in a flexion pattern; and (f) the center of gravity is not easily shifted to the right when walking (1).

The incidence of PS in stroke patients is 10% (2). Patients with PS have significantly lower balance, walking ability, and activities of daily living (ADL) than those without (3). Studies have demonstrated that PS slows the recovery of ADL in patients and prolongs hospital stays (4).

The pathogenesis of PS is complex, and there are several perspectives, including subjective postural vertical (5), subjective visual vertical (6), and second graviceptive system (7). Many studies have shown (8–10) that PS is associated with deficits in higher spatial processing, which may explain why patients with right brain lesions show more severe tilts to the contralateral side (7). Therefore, patients with spatial cognition deficits or visual–spatial sensory integration deficits after stroke may present with severe balance dysfunction.

The recovery of PS is slow, and the course of treatment is long (11). Visual feedback (12, 13), head and neck postural control, stimulation of hypotonic lateral trunk flexor activity, induction and strengthening of the affected lower limb extensors, and weight shifting are the usual treatment methods for patients with balance disorders (12, 14). Yang et al. (12) found that computer-simulated balance task training offered significant improvements in patients compared with conventional visual feedback therapy with objective objects. Dutta et al. (15) found that noninvasive brain stimulation combined with postural correction reduced functional impairment in PS patients. Finally, Pardo et al. (16) showed that treatment with limb transfer retraining, midline perception, and neurological re-education can improve balance in PS patients in roughly 4 weeks.

Therefore, finding an effective treatment remains the focus of rehabilitation experts. In the current study, two patients with PS combined with hemispatial neglect after basal ganglia stroke were treated with visual deprivation on the hemiplegic side and achieved rapid and remarkable results.

## Case Descriptions

### Patient 1

**Figure 1:**
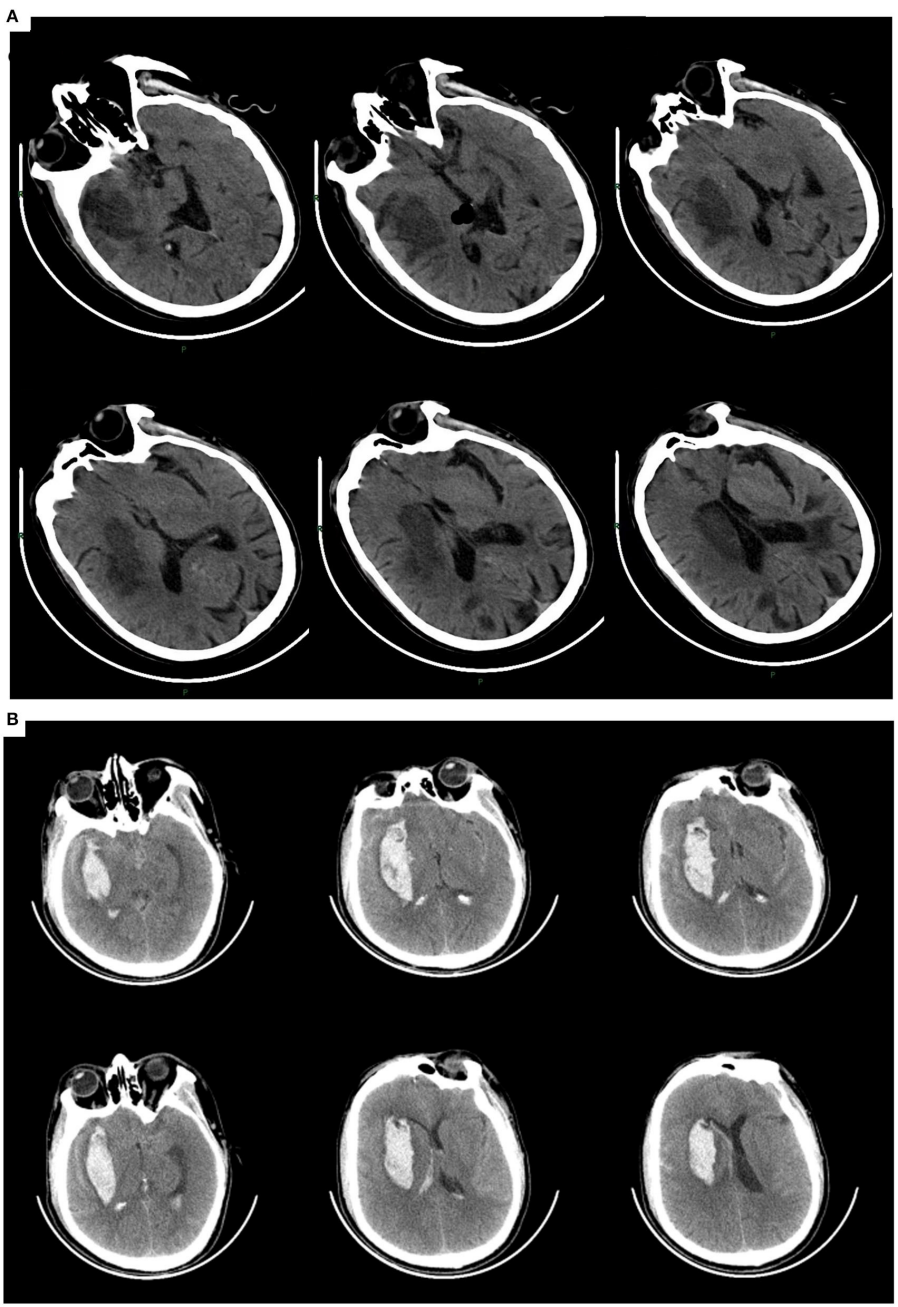
**(A)** Patient 1. Cerebral infarction in the right basal ganglia involving the temporal lobe, with a previous infarction in the left occipital lobe. **(B)** Patient 2. Intracerebral hemorrhage in the right basal ganglia involving the temporal lobe.

A 65-year-old male farmer was admitted to the hospital 17 days after a cerebral infarction. He presented with a severe tilt to the left and was unable to sit or stand unaided. He had a history of hypertension of more than 10 years and left occipital lobe infarction 5 years prior without sequelae after recovery. Upon physical examination, Patient 1 demonstrated normal levels of consciousness and cooperation. He completed three steps of the listening comprehensive test with no obvious abnormalities in comprehension; however, he had a decreased memory and calculation ability. There were no obvious abnormalities in comprehension, visual field, or hemianopia, but disorientation was present. His Mini-Mental State Examination (MMSE) score, which was valued at 24 with a junior middle school education level, indicated normal cognition. Both eyes rotated right when the head turned right. Notably, his ability to perceive stimuli on the left side was significantly reduced, as the patient tilted to the left severely when sitting down. He would resist strongly with attempts to hold him in a neutral position, which caused profound difficulty in transferring because of the strong reverse pushing from the right limbs. In the standing position, he could not maintain balance even with assistance and tilted to the left while sitting. The deep and superficial sense of his left limbs became extinct. The modified Ashworth scale score of his left limb was 0. The myodynamia of the right limbs was normal (5/5), while both the upper and lower left limbs were 3/5. The Babinski sign was positive on the left side. Left hemispatial neglect was assessed using line bisection, number cancelation, and copy drawing tests (17, 18). The Burke Lateropulsion Scale (BLS) (19) was 12. The arm–hand–leg Brunnstrom assessment of his left side was III–III–III. The Fugl–Meyer Balance Scale (FMBS) was 0. The Holden walking ability grade (HG) was 0, indicating that he could not walk. The modified Barthel ADL index (BI) was 23, indicating severe functional impairment. Brain computed tomography revealed an infarction in the right basal ganglia. His diagnoses included basal ganglia infarction, PS, left hemispatial neglect, and hypertension (Figure 1).

### Patient 2

A 55-year-old male farmer with a 5-year history of hypertension was admitted to the hospital 24 days after a cerebral hemorrhage. He also presented with left hemiplegia with a severe leftward tilt. Upon arrival, the patient was conscious and cooperative on physical examination with a normal visual field and no hemianopia. He could speak fluently without dysarthria and completed three steps of the listening comprehension test. His MMSE score was 24, with an educational level of junior high school. When he turned his head and neck to the right, both eyes rotated right. His ability to receive stimuli from the left side was significantly decreased, and both sitting and standing balance could not be maintained. When standing with assistance, the patient tilted to the left severely, and his posture was askew as his barycenter moved to the left. The flexion state of the right lower limb made it difficult to bear weight while standing. He resisted strongly when pushed rightward to the neutral position. The deep and superficial senses of the left limbs were decreased. The arm–hand-leg Brunnstrom assessment of the left side was II–II–III. The mean HG was 0. The modified BI score was 25, indicating severe functional impairment. The FMBS and BLS were 0 and 13, respectively. Left hemispatial neglect was revealed by the line bisection and number cancelation tests. The patient's muscle strength in his left arm was 1/5, and the strength in his left leg was 2/5, while the myodynamia of the right limbs was 5/5. The modified Ashworth scale of the left limb was 0. A positive Babinski sign was observed on the left side. His diagnoses were intracerebral hemorrhage in the basal ganglia, PS, left hemispatial neglect, and hypertension (Figure 1).

## Diagnostic Assessments

### Patient 1

**Figure 2:**
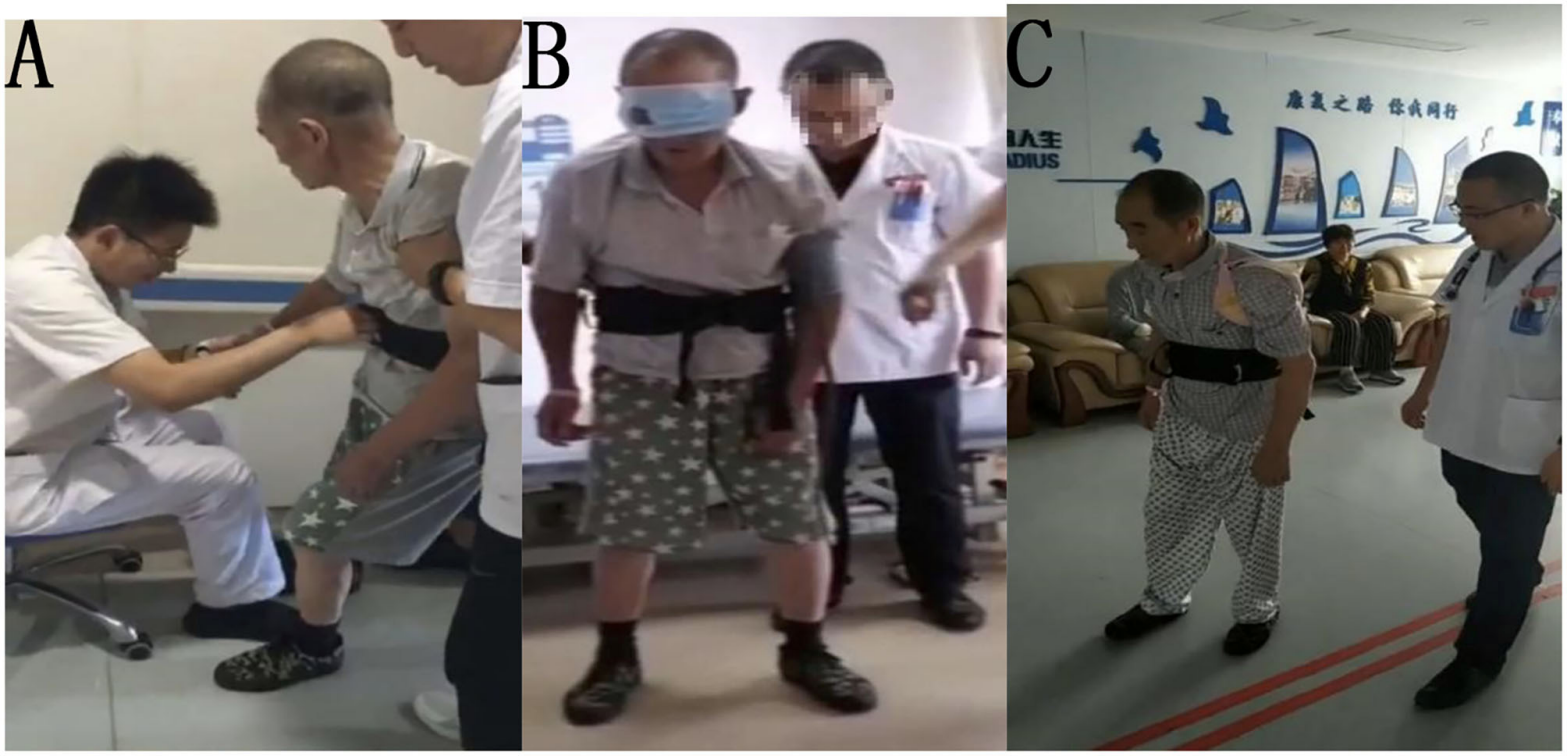
**(A)** Before treatment, **(B)** during treatment, **(C)** after treatment.

The patient was treated for 10 days with conventional rehabilitation methods (40 min, twice per day), including visual feedback, head and neck control, torso control, adjustment of midline perception, induction and enhancement of extensor muscles of the affected lower limb, and shifting of the barycenter. After treatment, the performance of the patient was evaluated and showed that the sitting tilt angle decreased, and the resistance of the patient to correction was also reduced; however, the patient was still unable to stand and walk. Then we guided the patient to use his right elbow and forearm to support himself while sitting, with the gravity center of his body significantly shifted to the right. As he gradually standing up, he was asked to straighten the right arm and support himself on the bed with his right hand. After repeating this vertical transfer several times, we covered his left eye completely and found that he could immediately transfer vertically by himself and maintaining standing position for several seconds. Following this, the patient continued to apply left visual deprivation for 30 min, twice per day. After 1 week of training, the patient could walk indoors under supervision for a short distance without visual deprivation. His BLS improved to 2, FMBS improved to 11, HG improved to 3 (i.e., walking under supervision), and BI score improved to 70 (Figure 2). At the time of discharge after 4 weeks in the hospital, the patient was able to walk approximately 150 m continuously indoors, but still required supervision.

### Patient 2

**Figure 3:**
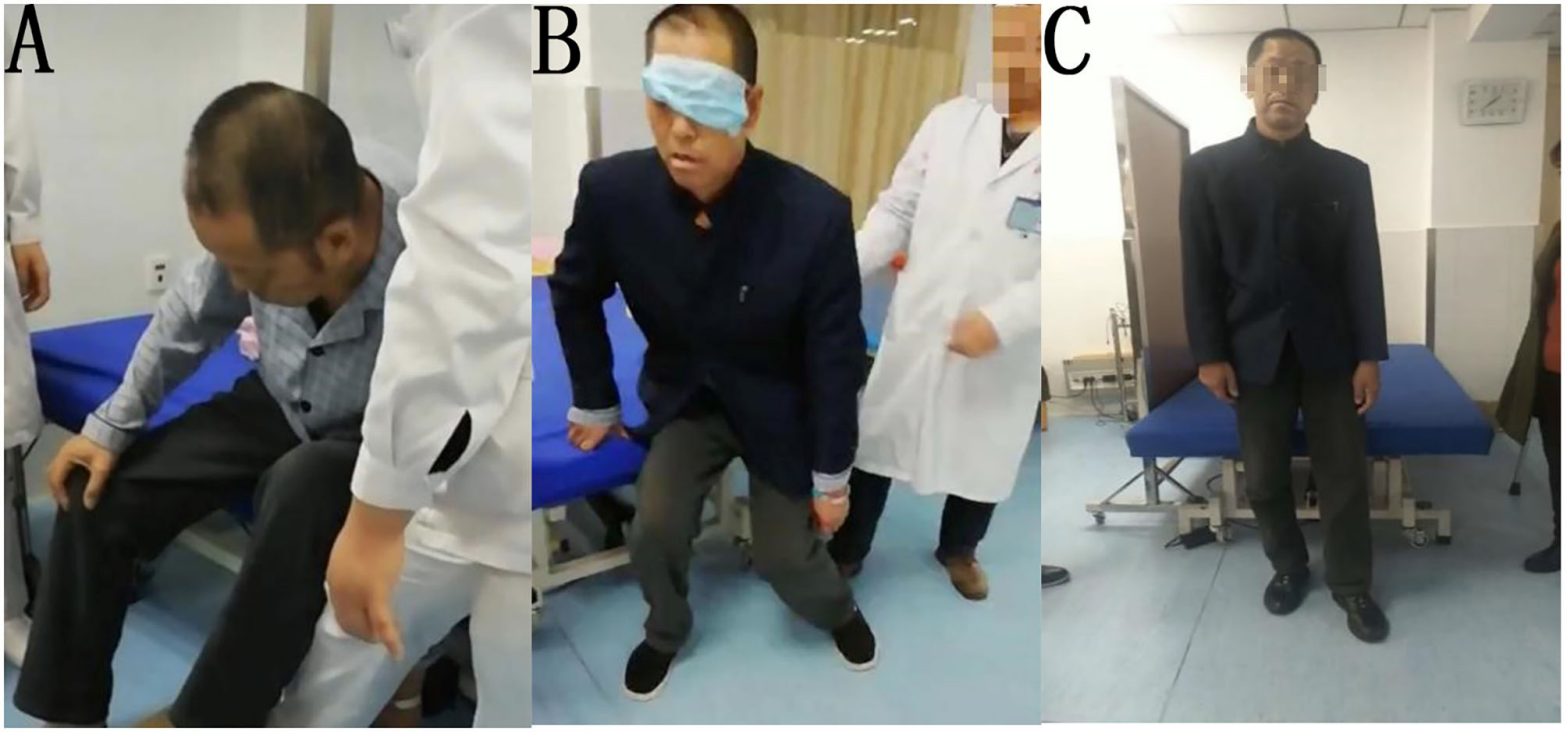
**(A)** Before treatment, **(B)** during treatment, **(C)** after treatment.

The patient was treated for 1 week with the same rehabilitation treatments as Patient 1. The tilt angle of the patient decreased slightly while sitting, but he could not establish balance while sitting, standing, or walking. After vision deprivation training, the patient could stand independently and maintain balance momentarily. We used the same method as Patient 1 to train the standing and walking ability of the patient for 30 min, twice per day. The patient could also immediately maintain standing position for several seconds after we covered his left eye. After 1 week of treatment, the patient could walk under supervision for a short distance indoors without visual deprivation. His BLS improved to 3, FMBS improved to 10, HG improved to 3 (i.e., walking under supervision), and BI improved to 60 (Figure 3). At the time of discharge after 4 weeks of treatment in total, the patient was able to walk approximately 200 m continuously indoors, but still required supervision.

## Discussion

Karnath et al. (7) showed that the etiology of PS, vascular distribution area, and size of the damage were highly correlated with neurological deficits. Several studies have found that brain regions such as the posterior and lateral ventral nuclei of the thalamus, insular cortex, and part of the parietal lobe (20, 21) are associated with PS pathogenesis. More recent studies have shown that a coherent cortico-subcortical network (i.e., basal ganglia and temporal lobe) comprising the right superior temporal gyrus, putamen, and caudate nucleus performs the cognitive processing functions of spatial perception and consciousness (22). The clinical manifestations of PS are extremely complex as lesions in each of these brain regions can cause corresponding PS. Among them, the superior temporal gyrus is the primary site for performing spatial perception. PS with lesions involving the superior temporal gyrus will result in a decrease in visuospatial processing (22, 23). The injury sites of our two patients in the current study were the basal ganglia region (i.e., putamen) and temporal lobe.

Unilateral spatial neglect (USN) is an attention-arousal deficit caused by damage to cortical sensory processing pathways and is most often seen in the right hemisphere injuries (24, 25). Patients present with a loss of spatial attention to the hemiplegic side and are unable to perceive or respond correctly. In a retrospective analysis of a large sample, Dai et al. (10) found that all patients with PS had USN and 97% of those with USN had PS. Chen et al. (26) reported that PS correlated with USN in approximately 80% of patients with right hemispheric injury.

Dieterich et al. (27) suggested that the onset of PS is differentiated between the left and right hemispheres and that spatial orientation, memory, and navigation are mainly governed by the right side of the cerebral cortex. Right-sided brain lesions exhibit a more severe tilt to the affected side compared with left-sided brain lesions, especially in patients with spatial cognition deficits or visual–spatial sensory integration deficits (8). Studies have found (28, 29) that each hemisphere has its own reticular–limbic system–cortical pathway, but that the left hemisphere of the brain attends only to stimuli from the contralateral space (i.e., right side), whereas the right hemisphere attends to stimuli from both sides of the space; therefore, the right hemisphere is considered the dominant hemisphere for spatial attentional control. Moreover, right hemisphere brain damage can lead to USN on the left side. Many theories interpret the mechanisms of hemispatial neglect, including interhemispheric competition (30, 31). This theory suggests that when one hemisphere of the brain is damaged (commonly the right side), there is an impairment of orienting attention and visual information representation in the contralateral space. Additionally, the damaged hemisphere has diminished inhibitory effects on the contralateral hemisphere, which results in an over activation of the corresponding functions in the contralateral hemisphere, producing a strong tendency to pay attention to the right and ignore the left.

The aforementioned studies suggest that PS is associated with lateralized visuospatial neglect, and therefore, it is inferred that disrupted higher cognitive processes in the visual pathway have an important role in the development of PS. Considering that PS associated with lateral spatial neglect is based on the spatial perception of visual information afferents, new treatment options should be investigated from visual control interventions (32).

Based on the above, we theorize that if the stroke is within the right basal ganglia and superior temporal gyrus, this would cause a bilateral imbalance in the perception of visuospatial information, leading to a left lateral spatial neglect and corresponding over activity of the left hemisphere (with increased excitability of the corresponding motor cortical areas), resulting in PS. By masking the left eye, treatment reduces the input of right-sided visuospatial sensory information and promotes the balance of spatial perception on both sides, thus, reducing the excitability of the corresponding brain areas in the left hemisphere and the tendency to pay attention to the right side. This also reduces the excitability of the posterior parietal and motor cortical connections in the left hemisphere, therefore, improving the “pushing phenomenon” and corresponding motor functions in PS patients.

Both patients were unable to balance while sitting on admission and establish stable balance while seated after 1 week of initial conventional rehabilitation (i.e., visual guidance, weight transfer, improving muscle tone on the paralyzed side of the trunk, and perceptual training). With the use of visual deprivation therapy, both patients were able to achieve standing balance within a few minutes and were able to walk short distances after 1 week. This suggests that visual deprivation therapy is highly effective for these patients. Compared with traditional visual feedback-based treatment, both patients were treated with visual deprivation therapy, which had a rapid effect and improved their ability to care for themselves. Due to the small sample number, more patients need to be observed to further verify the efficacy of the treatment. The efficacy of visual deprivation training for PS due to other areas of brain injury should be further investigated. Further evaluation of the functional areas described in the current study using functional imaging techniques is also a direction for our future research.

## Data Availability Statement

The original contributions presented in the study are included in the article/Supplementary Material, further inquiries can be directed to the corresponding authors.

## Ethics Statement

The studies involving human participants were reviewed and approved by Ethics Committee of The Geriatric Hospital Affiliated to Nanjing Medical University. The patients/participants provided their written informed consent to participate in this study. Written informed consent was obtained from the individual(s) for the publication of any potentially identifiable images or data included in this article.

## Author Contributions

LZ and QZ designed and conceptualized the study. LZ, QZ, ZZ, JZha, and SC drafted the manuscript. WH, XZhe, and HX created the therapeutic intervention. WJ and CZ collected the information. JZhe and XZhu organized the data. YL, SX, and SC analyzed the data. All authors approved the final version of the manuscript.

## Conflict of Interest

The authors declare that the research was conducted in the absence of any commercial or financial relationships that could be construed as a potential conflict of interest.

## Publisher's Note

All claims expressed in this article are solely those of the authors and do not necessarily represent those of their affiliated organizations, or those of the publisher, the editors and the reviewers. Any product that may be evaluated in this article, or claim that may be made by its manufacturer, is not guaranteed or endorsed by the publisher.
