# Supplementary material for: Case Report: Visual Deprivation in Pusher Syndrome Complicated by Hemispatial Neglect After Basal Ganglia Stroke
Source: Front Neurol. 2021 Sep 22;12:706611. doi: 10.3389/fneur.2021.706611 (PMC8494178; doi:10.3389/fneur.2021.706611)
Supplement: Supplementary file 1 [file Data_Sheet_1.PDF]

Video description

Four videos for Patient 1

Video 1: before visual deprivation

Video 2: after visual deprivation, recorded just after video 1

Video 3: indoor walking (after 1 week of visual deprivation training)

Video 4: indoor walking (2 weeks after leaving the hospital)

One video for Patient 2

Video: vertical transfer by himself and walk for a short distance 1 week after visual deprivation training
